# Coxsackievirus A16 Encephalitis during Obinutuzumab Therapy, Belgium, 2013

**DOI:** 10.3201/eid2005.131766

**Published:** 2014-05

**Authors:** Tom Eyckmans, Elke Wollants, Ann Janssens, Hélène Schoemans, Katrien Lagrou, Joost Wauters, Johan Maertens

**Affiliations:** University Hospitals Leuven, Leuven, Belgium

**Keywords:** coxsackievirus, coxsackievirus A16, enterovirus, viruses, encephalitis, monoclonal antibody, obinutuzumab, CD20 B-cell antigen, Belgium

**To the Editor:** Enterovirus infections are associated with many clinical manifestations, and specific virus groups or serotypes are associated with specific manifestations. Coxsackievirus A16, a common cause of hand, foot and mouth disease, rarely causes encephalitis. Although most enterovirus infections are cleared by cellular immune responses, invasive enterovirus disease is prevented or controlled by neutralizing antibodies (*1*). Thus, patients with humoral immunodeficiencies are susceptible to serious enterovirus infections.

Nine cases of enteroviral encephalitis (1 caused by echovirus 13, 1 caused by coxsackievirus A16, 2 caused by enterovirus 71, and 5 caused by unknown enteroviruses) have been reported after therapy with rituximab, a monoclonal antibody (MAb) that causes secondary hypogammaglobulinemia (*2*). We describe coxsackievirus A16 encephalitis in a patient who was receiving treatment with the MAb obinutuzumab.

A 67-year-old woman with non-Hodgkin lymphoma showed complete remission after 6 cycles of treatment with bendamustine and obinutuzumab. Induction immunochemotherapy was followed by obinutuzumab maintenance therapy. At admission, she had received 7 of 12 scheduled treatments.

The patient was hospitalized because of a history of high-grade fever that did not respond to antimicrobial drugs, confusion, general weakness, and urinary incontinence. She had a neutrophil count of 3.1 × 10^9^ cells/L but had severe lymphocytopenia (0.3 × 10^9^ cells/L and an absolute CD4 cell count of 0.082 × 10^9 ^cells/L) and low serum immunoglobulin levels (IgG 3.86 g/L [reference range 7.51–15.6 g/L], IgA 0.07 g/L [reference range 0.82–4.53 g/L], and IgM 0.13 g/L [reference range 0.46–3.04 g/L]). Cerebrospinal fluid (CSF) samples were collected on days 1, 4, and 6. CSF leukocyte counts increased from 14 cells/mm^3^ (day 1) to 60 cells/mm^3^ (day 4) (35% and 27% lymphocytes, respectively). Cytologic and immunophenotypic analyses showed no cerebromeningeal lymphoma infiltration. Total protein levels in CSF increased from 561 mg/L on day 1 to 771 mg/L on days 4 and 6.

Bacterial and fungal cultures were negative. Cryptococcal antigen was not detected in CSF. Serologic test results were negative for *Borrelia* spp., *Listeria* spp., parvovirus B19, measles virus, and galactomannan. PCR results for CSF were repeatedly negative for herpes simplex virus, varicella zoster virus, cytomegalovirus, *Toxoplasma gondii*, JC polyomavirus, human herpesvirus 6, Epstein-Barr virus, and mumps virus. PCR results and culture were negative for *Mycobacterium* spp. Serum samples were negative for antibodies against neuronal nuclear (Hu, Ri, and Yo) antigens. However, enterovirus RNA was detected by reverse transcription PCR in all CSF samples. Sequencing of the virion protein 1 gene obtained directly from RNA extracted from CSF identified the virus as coxsackievirus A16 (*3*).

Computed tomography scan of the brain on day 2 showed no abnormalities. However, brain magnetic resonance imaging scans on the third and fourth days showed bilateral, multiple, hyperintense white matter lesions in the periventricular region and cerebral hemispheres. Treatment was started empirically with broad-spectrum antimicrobial drugs and acyclovir; the acyclovir was stopped after infection with herpes simplex virus was excluded.

On day 4, imaging indicated development of aphasia and right hemiparesis without new lesions. The patient was transferred for mechanical ventilation after a grand mal seizure. Treatment with intravenous immune globulin (IVIG, 400 mg/kg) was started on day 4 and given for 5 consecutive days, which resulted in marked and continued neurologic improvement. Monthly doses of IVIG (500 mg/kg) resulted in normal serum IgG levels. Four months after initial examination, the virus has been cleared but the patient still has intermittent confusion and language defects. Treatment with IVIG will be continued for an additional 6 months.

Use of MAbs against CD20 B-cell antigen has become standard treatment for B-cell lymphomas and an increasing number of autoimmune disorders (*4*,*5*). However, resulting hypogammaglobulinemia predisposes patients to opportunistic infections, including progressive multifocal leukoencephalopathy and enterovirus infections (*2*,*6*). MAbs with enhanced activity against CD20 (e.g., obinutuzumab) have been developed. Obinutuzumab has been approved by the US Food and Drug Administration for treatment of chronic lymphocytic leukemia. Studies regarding the use of obinutuzumab for other B-cell malignancies are ongoing (*7*).

We anticipate that more cases of enteroviral encephalitis might develop, given the increasingly frequent use of MAbs against CD20 and widespread occurrence of enteroviruses. However, in view of the few reported cases (*2*), we also suspect that many cases remain undiagnosed despite availability of several pan-enterovirus diagnostic kits, which can detect low viral loads. Thus, clinicians should be suspicious of severe enterovirus infections in patients receiving MAbs.

Any patient receiving MAbs against CD20 who has neurologic symptoms should be screened for infection with enterovirus RNA. In contrast to JC polyomavirus–associated progressive multifocal leukoencephalopathy, enteroviral encephalitis can be successfully treated by early administration of IVIG, which might contain neutralizing antibodies, albeit in variable amounts (*8*). In the absence of double-blinded, placebo-controlled clinical studies of treatment for severe enterovirus infections, no specific antiviral therapy has been approved. However, 3 capsid inhibitors (pleconaril, pocapavir [V-073], and the pirodavir analog BTA-798) that show activity against enteroviruses are being developed (*9*). Pocapavir has potent activity against poliovirus and appears to be safe and well tolerated (*10*). In the United States, this drug is available by special request from the Food and Drug Administration.

## References

[1] Mao Q, Wang Y, Yao X, Bian L, Wu X, Xu M, Coxsackievirus A16: epidemiology, diagnosis, and vaccine. [Epub ahead of print]. Hum Vaccin Immunother. 2013;10. [Epub ahead of print]. 10.4161/hv.27087 PMC4185891 24231751

[2] Kassab S, Saghi T, Boyer A, Lafon ME, Gruson D, Lina B, Fatal case of enterovirus 71 infection and rituximab therapy, France, 2012. Emerg Infect Dis. 2013;19:1345–7. 10.3201/eid1908.130202 23880543 PMC3739532

[3] Thoelen I, Moës E, Lemey P, Mostmans S, Wollants E, Lindberg AM, Analysis of the serotype and genotype correlation of VP1 and the 5′ noncoding region in an epidemiological survey of the human enterovirus B species. J Clin Microbiol. 2004;42:963–71. 10.1128/JCM.42.3.963-971.2004 15004039 PMC356875

[4] Griffin MM, Morley N. Rituximab in the treatment of non-Hodgkin’s lymphoma: a critical evaluation of randomized controlled trials. Expert Opin Biol Ther. 2013;13:803–11 . 10.1517/14712598.2013.786698 23560506

[5] Rosman Z, Shoenfeld Y, Zandman-Goddard G. Biologic therapy for autoimmune diseases: an update. BMC Med. 2013;11:88. 10.1186/1741-7015-11-88 23557513 PMC3616818

[6] Casulo C, Maragulia J, Zelenetz AD. Incidence of hypogammaglobulinemia in patients receiving Rituximab and the use of intravenous immunoglobulin for recurrent infections. Clin Lymphoma Myeloma Leuk. 2013;13:106–11. 10.1016/j.clml.2012.11.011 PMC4035033 23276889

[7] Goede V, Fischer K, Busch R, Engelke A, Eichhorst B, Wendtner CM, Obinutuzumab plus chlorambucil in patients with CLL and coexisting conditions. N Engl J Med. 2014. [Epub ahead of print]. 10.1056/NEJMoa1313984 24401022

[8] Wildenbeest JG, van den Broek PJ, Benschop KS, Koen G, Wierenga PC, Vossen AC, Pleconaril revisited: clinical course of chronic enteroviral meningoencephalitis after treatment correlates with in vitro susceptibility. Antivir Ther. 2012;17:459–66 . 10.3851/IMP1936 22293148

[9] Abzug MJ. The Enteroviruses: problems in need of treatments. J Infect. 2014;68:S108–14 and. 10.1016/j.jinf.2013.09.020 24119825

[10] Hincks JR, Collet MS. Safety and pharmacokinetics of pocapavir, an oral antiviral candidate against poliovirus. In: Abstracts of the 53th Interscience Conference on Antimicrobial Agents and Chemotherapy, Denver, Colorado, Sep 10–13, 2013. Washington (DC): American Society for Microbiology; 2013. Abstract no. A-017d.

